# Molecular analysis of a large novel deletion causing α^+^-thalassemia

**DOI:** 10.1186/s12881-019-0797-8

**Published:** 2019-05-06

**Authors:** Jianlong Zhuang, Jie Tian, Jitao Wei, Yu Zheng, Qianmei Zhuang, Yuanbai Wang, Qingyue Xie, Shuhong Zeng, Geng Wang, Yanchao Pan, Yuying Jiang

**Affiliations:** 1 Prenatal Diagnosis Center, Quanzhou Woman’s and Children’s Hospital, No. 700 Fengze Street Fengze District, Quanzhou City, 362000 Fujian Province People’s Republic of China; 2 Yaneng BIOscience (Shenzhen) Co. Ltd, 518000 Shenzhen City, Guangdong Province People’s Republic of China; 3 Shishi Maternal and Child Health Hospital, 362700 Quanzhou City, Fujian Province People’s Republic of China

**Keywords:** α-Thalassemia, Gap-PCR, MLPA, Sequencing

## Abstract

**Background:**

α-thalassaemia is an inherited blood disorder caused by mutations in the α-globin gene cluster. Recognizing the pathogenic α-globin gene mutations associated with α-Thalassemia is of significant importance to thalassaemia’s diagnosis and management.

**Methods:**

A family with α-thalassaemia from Fujian, China was recruited for this study. The phenotype was confirmed through haematological analysis. Commercially available Gap-PCR genotypic methods were employed to identify the known deletions causing α-thalassemia. MLPA analysis was used to study the novel mutations; this was then confirmed through DNA sequencing and bioinformatics analysis.

**Results:**

The proband of the family belonged to Southeast Asian type (--^SEA^) thalassaemia. None of the known mutations associated with α-thalassaemia were detected in this family’s genetics, whereas a novel 6.9 kb deletion (16p13.3 g.29,785-36,746) covering the α2 gene on the globin gene cluster was identified with MLPA and confirmed through Sanger Sequencing. This data led us to propose a novel pathogenic deletion associated with α-thalassemia: -α^6.9^ /--^SEA^.

**Conclusions:**

A novel α-thalassaemia deletion was identified in members of a Chinese family and subsequently analyzed. This finding has helped broaden the spectrum of pathogenic mutations leading to the development of α-thalassaemia, paving the way for improved disease diagnosis and management.

## Background

Approximately 5% of the world’s population carries globin gene mutations, of which 1.7% exhibit symptoms of α-thalassaemia [1–3]. α-thalassaemia is one of the world’s most common hemoglobin disorders associated with deletion or point mutations in α-globin genes clusters. The cluster responsible for this disease is about 30 kb in size and located on the end of chromosome 16p13.3 in the order of 5’-ζ-ψζ-ψα1-α2-αl-θ-3’. Southeast Asian deletion (--^SEA^), right deletion (-α^3.7^) and left deletion (-α^4.2^) are the top three deletions found responsible for α-thalassaemia, whereas Hb ConstantSpring (HBA2:C.427T>C), Hb QuongSze (HBA2:c.377T>C) and Hb Westmead (HBA2:c.369C>G) are the predominant non-deletion types discovered to date. The --^SEA^, -α^3.7^, -α^4.2^, Hb CS and Hb QS account for about 90% of mutations in Chinese populations [4]. In addition, rare deletions including --^JS^, --^11.1^, --^FIL^, --^THAI^, -α^27.6^, -α^21.9^, -α^2.4^, -α^3.8^ and -α^2.8^ have been reported as being associated with α-thalassaemia [5–16]. Gap-PCR is used to diagnose α-thalassaemia associated with these rare mutations. Multiple ligation-dependent probe amplification (MLPA) or next generation sequencing (NGS) technologies can be employed to characterize unknown mutations at the molecular level.

In this study, we investigated a Chinese family carrying α-thalassaemia and examined the molecular causes underlying the family’s phenotype. An associated novel pathogenic deletion was discovered and characterized for the first time. The proband showed a decrease of MCV and MCH levels and abnormal increase of HbH and HbBart’s levels. Thus we assume it can be an HbH disease. Finally, our results showed a novel deletional α-thalassemia range (NG-000006.1:g.29785-36746 del 6962bp) which covered all α2 gene but not α1 gene. The novel deletion of α-thalassemia also compound with --^SEA^ deletion type, thus the genotype of the proband can described as -α^6.9^/--^SEA^.

## Methods

### Patients

The proband, a 36 year old male of Han nationality, and his family, were recruited for this study. The couple screened positive for α-thalassaemia. Peripheral blood samples from the couple and their son were collected and stored for further investigation.

### Hematological analysis

A routine blood analysis was performed using an automated cell counter (Sysmex XS-1000i; Sysmex Co., Ltd., Kobe, Japan). The subjects’ levels of Hb A, Hb A2 and Hb F were detected with hemoglobin capillary electrophoresis (Sebia, Evry Cedex, France).

### Molecular analysis

The family members’ genomic DNA was obtained at Ruibao Biological Co., Ltd. using an automatic nucleic acid extractor. The gene deletion mutation analysis of α-thalassaemia (-α^3.7^, -α^4.2^, --^SEA^) was performed using gap-PCR [17–19]. The PCR reverse dot hybridization technique (PCR-RDB) was used to diagnose the non-deletion α-thalassaemia (Hb CS, Hb QS and Hb Westmead), and 17 common mutations were found and associated with β-thalassaemia, [20–22] including CD41–42 (−TCTT), IVS-II-654 (C>T), –28 (A > G), CD 71/72 (+A), CD 17 (AAG > TAG), CD 26 (GAG>AAG), CD43 (GAG>TAG), –29(A > G), CD31 (−C), –32 (C > A), IVS-I-1 (G > T), CD 27/28 (+C), –30(T > C), CD 14/15 (+G), Cap+ 40–43 (−AAAC), initiation codon (ATG > AGG) and IVS-I-5 (G > C). Six α-thalassaemia genotype-screening kits (Yaneng Biological technology Co., Ltd., Shenzhen) were utilized to detect --^THAI^, -α^27.6^, HKαα, fusion gene, ααα^anti4.2^ and ααα^anti3.7^. In order to detect unknown variants, a multiplex ligation-dependent probe amplification (MLPA) assay was employed using the SALSA MLPA probemix P140-C1HBA (MRC-Holland, Amsterdam, Netherlands).

### DNA sequencing and analysis

Gap-PCR was used to identify the deletion breakpoints. According to the known DNA sequences around the breakpoints, specific primers were designed. These primer sequences were P1: GGAGAACTTGGCCCCACGTTATCTA and P2: GGCGCTGTCGGCTCGTGCA. All primers were synthesized at Invitrogen (Shanghai, China). Gap-PCR reaction system: 2 mmol dNTP 2 μL, 5 × buffer 4 μL, 25 mmol MgCl_2_ 2 μL, Taq enzyme 1 U, 10 μmol primers 0.5 μL each, template 2 μL, and plus ultra-pure water to 20 μL. Amplification conditions: 96 °C for 5 min; 98 °C for 30 s, 60 °C for 1 min, 72 °C for 2 min, 35 cycles; and 72 °C for 10 min. Electrophoresis analysis was performed and the purified electrophoresis products then sent for Sanger sequencing. The sequenced data were analyzed with GenBank NG_000006.1 as their reference sequences.

## Results

### Hematological analysis

As shown in Table 1, the MCV, MCH and Hb A2 levels of the proband and his son were much lower than that of the normal reference. In addition, hemoglobin capillary electrophoresis data revealed that the red cells of the proband contained 14% of the HbH variant and 0.6% of Hb Bart’s variant. Thus we assumed the proband could be a patient with HbH disease. The proband’s wife’s Hb A2 level was 2.5%, being slightly lower than the normal content, while both her levels of MCV and MCH were normal. These combined observations allowed us to confirm that the proband and his family’s α-thalassemia phenotype.

**Table 1:** The hematological analysis of the proband, his wife and son

Parameters	Proband	Proband’s wife	Proband’s son
RBC(10^12^/L)	5.29	4.23	5.64
Hb(g/L)	111	127	112
Hct	34.8	37.6	35
MCV (fl)	65.8	88.9	62.1
MCH (pg)	21	30	19.9
Hb A(%)	84.6	97.5	97.6
Hb A2(%)	0.8	2.5	2.4
Hb H(%)	14	0	0
Hb Bart’s(%)	0.6	0	0

### Mutation analysis of the thalassemia genes

The common pathogenic mutations were screened using PCR-RDB, but no common point mutations for α-thalassemia or β-thalassaemia were found in the entire family, leading us to further investigate the causes underlying the proband’s symptoms.

**Fig. 1:**
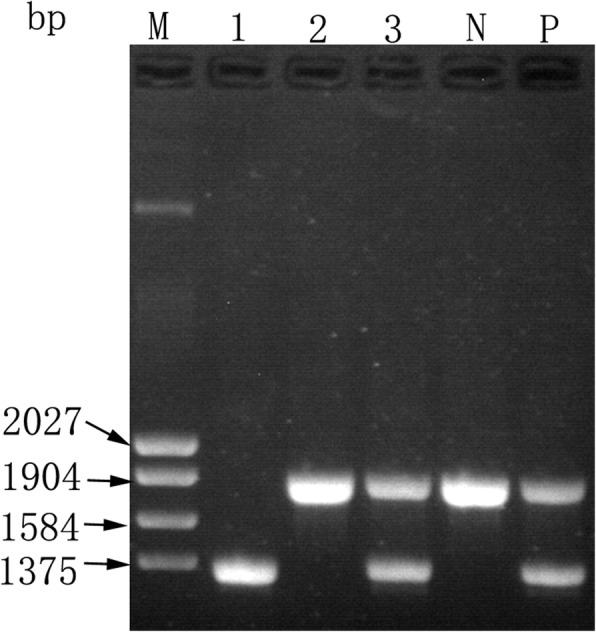
Common deletion type α-Thalassemia electrophoresis results.1: Proband. 2: Proband’s wife. 3: Proband’s son. N: Negative control. P: Positive control

A Gap-PCR analysis to detect three common α-thalassaemia deletions (-α^3.7^, -α^4.2^, --^SEA^) was performed, which revealed the presence of --^SEA^ in both the proband and his son. As shown in Fig. 1, only an 1826bp band, representing the normal gene, appeared in the proband’s wife’s genome. However, a 1306bp band, indicating the --^SEA^ mutation, was observed in the proband, and their son carried both bands. The Gap-PCR results suggest that the family’s genotypes are: (--^SEA^/--^SEA^, β^N^/β^N^) for the proband, (--^SEA^/αα, β^N^/β^N^) for his son and (αα/αα, β^N^/β^N^) for his wife.

To determine whether the mutation carried by the proband is a reported rare mutation, six α-thalassaemia genotyping kits were used to detect --^THAI^, -α^27.6^, HKαα, fusion gene [23], ααα^anti4.2^ and ααα^anti3.7^. However, none of these thalassaemia genes were found in the family genomes, suggesting that this may be a novel mutation.

### Novel mutations detection with MLPA and gap-PCR

**Fig. 2:**
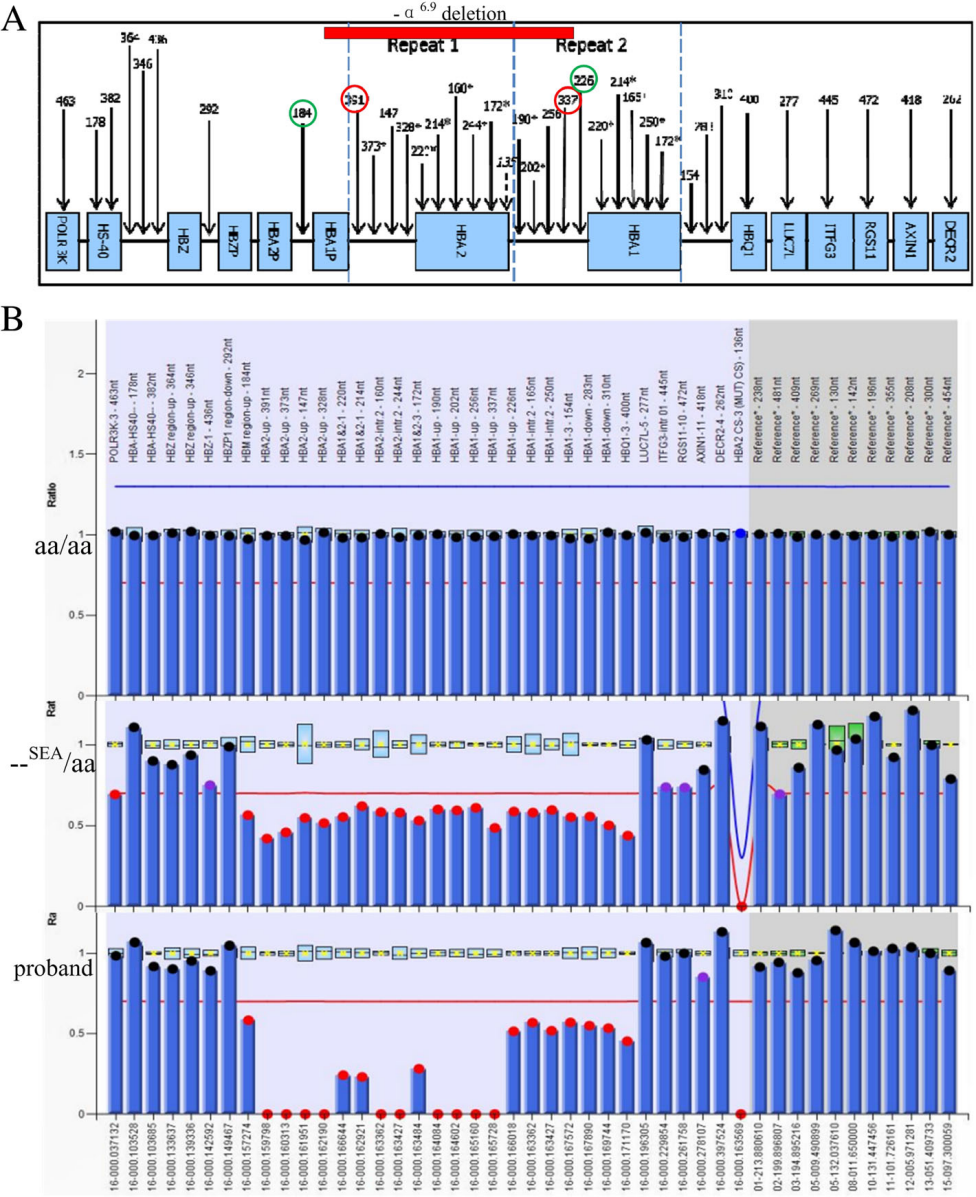
Multiplex ligation-dependent probe amplification assay results. **a** The corresponding position of amplification products (number unit in bp) and α-globin gene cluster (NG_000006.1) sequence in the P140-C1 HBA kit (MRC-Holland). **b** The corresponding fragment ratio of MLPA results from the αα/αα, --^SEA^/αα and proband. X-axis is the product fragment length (bp), while Y-axis represents their ratios. According to the manufacturer’s instructions, probe ratios between 0.7 and 1.3 are defined as normal

**Fig. 3:**
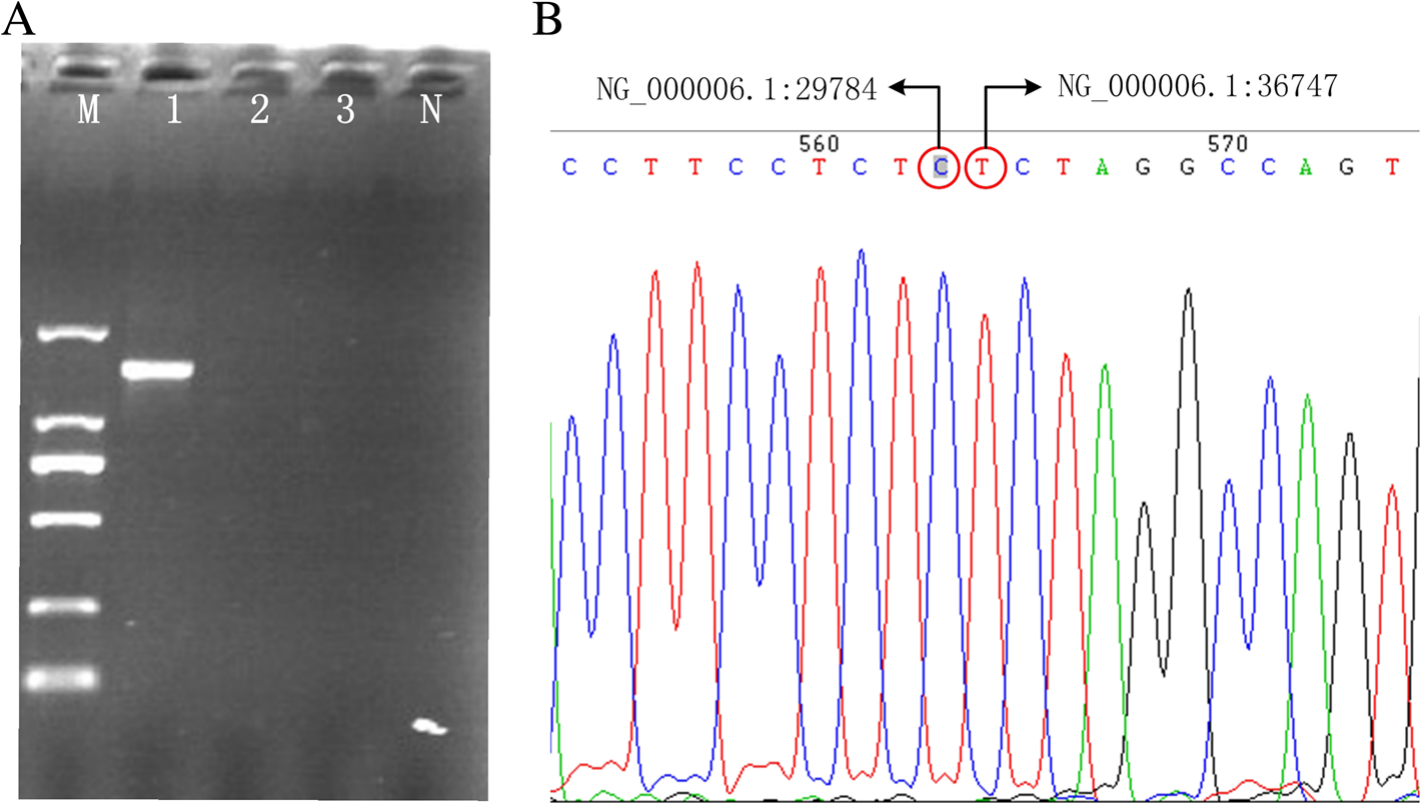
Polymerase chain reaction and sequencing analysis. **a** The PCR products were generated from the proband using primers P1 and P2. A unique 1.4 kb product was amplified in the sample of the proband, whereas DNA from Proband’s wife and son, and the normal individuals all failed to amplify this abnormal fragment. The 1.4 kb product was sequenced to determine the precise breakpoint of this deletion. M: DL2000 DNA marker. 1: Proband. 2: Proband’s wife. 3: Proband’s son. N: normal individuals. **b** Characterization of the breakpoints of this novel deletion by direct sequencing. Sequence analysis of the amplification obtained with gap-PCR using primers P1 and P2 helped us to characterize the breakpoints. As shown in the figure, a 6962bp sequence deletion (NG_000006.1: g.29785_36746) existed in the novel deletion

To further define the proband’s α-thalassaemia gene mutation, we used an MLPA assay for the DNA screening. As shown in Fig. 2, a large novel deletion in the α-globin gene cluster was detected. To confirm the results, we then analyzed the gene copy number deletion with two pairs of probes in MLPA. Probe Pair 1 consisted of Probes 184 (NG_000006.1: 28168-28169) and 226 (NG_000006.1: 36908-36909). Probe Pair 2 consisted of Probes 391 (NG_000006.1: 30716-30717) and 337 (NG_000006.1: 36628-36629). We discovered that the product ratio from Probe Pair 1 was 0.5, and from Pair 2 was 0. Because the proband also combined with --^SEA^, the 5’ breakpoint of this novel deletion should be localized at a 2.5 kb region between Probes 184 and 391. The 3’ breakpoint was delimited by a combination of Probes 337 and 226, yielding a 0.3 kb fragment. Gap-PCR was performed with a pair of primers (P1 and P2) to confirm the locations of the breakpoints. Using normal human DNA such as the wife’s as the PCR template meant that the size of the gene product between the primers was too large to require amplification, whereas a 1.4 kb PCR product was observed with the proband’s DNA as the PCR template, indicating that the breakpoint of the deletion was located between the two primers. However, this 1.4 kb fragment was not observed in the son, suggesting that the son had not inherited the novel deletion and was merely a carrier of the --^SEA^ (Fig. 3a).

### DNA sequencing and analysis

To determine the exact location of the breakpoints, the 1.4 kb product of the gap-PCR was sequenced using the P1 and P2 Sanger sequencing primers. The obtained sequencing data were aligned with the reference sequence (NG_000006.1), using NCBI BLAST. The results demonstrated that this was a 6,962 bp deletion, ranging from g.29,785 to g.3,6746 (NG_000006.1: g.29,785-36,746 del 6,962 bp), and covering the α2 but not α1 gene (Fig. 3b). This was a novel deletion associated for the first time in reports with α-thalassaemia. Therefore, we named the new α-thalassaemia deletion -α^6.9^, with the thalassaemia genotype for the proband being described as -α^6.9^/--^SEA^, which can cause HbH disease.

## Discussion

α-thalassaemia is an inherited disorder caused with deletion or mutation events occurring on the α-globin chain. Based on different known combinations of these mutations, α-thalassaemia can be classified into four types, as follows: the silent carrier state (heterozygous to the α^+^ defect), α-thalassaemia minor (homozygous to the α^+^ or α^0^ defects), Hemoglobin H disease (HbH, compound heterozygous to the α^0^ and α^+^ defects) and Barts hydrops fetalis (homozygous to the α^0^ defect) [24, 25]. HbH disease usually produces less than 30% of the normal amount of α-globin due to a deletion of three genes (--/-α) [26–28]. The predominant features in HbH disease are anemia with variable amounts of HbH (0.8-40%), and occasionally accompanied by Hb Bart's syndrome in the peripheral blood [29].

The --^SEA^, -α^3.7^, -α^4.2^, Hb CS and Hb QS account for about 90% of the α-thalassaemia mutations in the Chinese population. In southern China, --^SEA^ was the most common α^0^ mutation, while -α^3.7^ and -α^4.2^ were the most common α^+^ mutation. The combination of the –^SEA^ deletion and these two α^+^ deletions led to the most common types of HbH disease associated with deletions [30, 31]. Most of the patients with -α^3.7^/--^SEA^ and -α^4.2^/--^SEA^ HbH disease developed mild and moderate anemia, and a few had no clinical symptoms [31]. In this study, we investigated a proband from a Chinese family who demonstrated mild anemia and a slight decrease in Hb, which is similar to the phenotypes of patients with -α^3.7^/--^SEA^ and -α^4.2^/--^SEA^ HbH disease [32]. Using a routine genotyping method developed based on known mutations or deletions, the genotypes of the family members were identified as (--^SEA^/--^SEA^, β^N^/β^N^) for the proband, (--^SEA^/αα, β^N^/β^N^) for his son and (αα/αα, β^N^/β^N^) for his wife, implying that the son is a minor carrier of --^SEA^ and the proband should be a severe Hydrops Fetalis patient. However, Hemoglobin Barts hydrops fetalis syndrome is generally a fatal clinical phenotype of α-thalassaemia observed in fetuses rather than adults. The inconsistency between the observed genotype and reported clinical phenotypes led us to deduce that an unknown or rare mutation may exist in this patient, whose mutations might be overlooked in routine genetic testing for known mutations of α-thalassaemia. By employing a combination of MLPA and gap-PCR analyses using custom primers, we located a previously unreported, novel 6.9 kb deletion (del16p13.3 g.29,785-36,746) on the patient’s α-globin chains. We named this deletion -α^6.9^, and we believe this is what led in combination with --^SEA^ to the development of HbH disease.

It is significantly important to recognize the pathogenic α-globin gene mutations associated with α-thalassaemia in thalassaemia disease diagnosis and management. The α-globin chains are encoded with two functional alpha genes (Alpha 1 and 2) located on the α-globin gene cluster [24]. This 6.9 kb deletion fragment in -α^6.9^/--^SEA^ overlaps with the deletions detected in -α^3.7^/--^SEA^ and -α^4.2^/--^SEA^ HbH patients, which could explain the observation that α^6.9^/--^SEA^, -α^3.7^/--^SEA^ and -α^4.2^/--^SEA^ HbH have similar phenotypes. Except for the different deletion sizes of -α^6.9^, -α^3.7^ and -α^4.2^, they all cover a single Alpha 2 gene, resulting in a decreased dosage of α-globin. Pathogenic mutations associated with one (α^+^ defects) or both (α^0^ defects) alpha genes (in *cis*) at the α-globin gene cluster can lead to malfunctions in alpha globin synthesis and metabolism.

Further to the discovery in our study of a novel pathogenic hotspot deletion associated with α-thalassaemia, it is important to realize that routine screening can lead to the omission of rare or novel pathogenic sites. To avoid false-negative results in thalassaemia diagnosis, hematological analysis and genetic testing should be applied strategically. When the results of routine genetic testing are inconsistent with the haematological analysis, detailed genetic testing should be carried out to determine the molecular causes for phenotypes. Our research has highlighted the importance of combining different technologies in achieving accurate diagnoses. Recently, next generation sequencing (NGS) technology has also provided a new strategy for genetic diseases screening, including that for thalassaemia. The application of next-generation sequencing to thalassaemia screening not only significantly reduces the possibility for obtaining false-negative results and misdiagnoses, but also eliminates the need for repeat blood sampling and further referral tests. NGS can be a potentially widespread screening method, especially among populations with a high prevalence of thalassaemia [30]; our group is further investigating this possibility.

## Conclusions

We identified a large, novel 6.9 kb deletion, covering α2 but not α1 and causing α-thalassemia. With a series validation and analysis, a new genotype, -α^6.9^/--^SEA^, of HbH disease is proposed. Our study expands upon the spectrum of pathogenic mutations associated with α-thalassaemia, thus improving clinical practice in the diagnosis and disease management of thalassaemia.

## Abbreviations

Hb: Hemoglobin
Hct: Red blood cell volume
MCH: Mean corpuscular hemoglobin
MCV: Mean corpuscular volume
MLPA: Multiplex ligation-dependent probe amplification
PCR-RDB: PCR reverse dot hybridization
RBC: Red blood cell count
